# Interpreting the Theranostic Applications of Alumina and Silica Substrates in Cancer

**DOI:** 10.3390/molecules31030428

**Published:** 2026-01-26

**Authors:** Dimitris-Foivos Thanos, Pavlos Pantelis, Giorgos Theocharous, Sylvia Vagena, Cleo Kyriakopoulou, Giannis Pantelidis, Mary Markatou, Myrto Pliakostamou, Nikolaos Papanikolaou, Ekaterina-Michaela Tomou, Maria-Anna Gatou, Evangelia A. Pavlatou, Natassa Pippa, Vassilis G. Gorgoulis, Nefeli Lagopati

**Affiliations:** 1 Molecular Carcinogenesis Group, Department of Histology and Embryology, Medical School, National and Kapodistrian University of Athens, 11527 Athens, Greece; 2 Vascular Unit, First Propedeutic Department of Surgery, National and Kapodistrian University of Athens, Hippocration Hospital, 11527 Athens, Greece; 3 Laboratory of Biology, Department of Basic Medical Sciences, Medical School, National and Kapodistrian University of Athens, 11527 Athens, Greece; 4 Laboratory of General Chemistry, School of Chemical Engineering, National Technical University of Athens, Zografou Campus, 15772 Athens, Greece; 5 Section of Pharmacognosy and Chemistry of Natural Products, Department of Pharmacy, School of Health Sciences, National and Kapodistrian University of Athens, 15784 Athens, Greece; 6 Section of Pharmaceutical Technology, Department of Pharmacy, School of Health Sciences, National and Kapodistrian University of Athens, 15771 Athens, Greece; 7 Division of Cancer Research, Ninewells Hospital and Medical School, University of Dundee, Dundee DD1 9SY, UK; 8 Biomedical Research Foundation, Academy of Athens, 11527 Athens, Greece; 9 Faculty Institute for Cancer Sciences, Manchester Academic Health Sciences Centre, University of Manchester, Manchester M20 4GJ, UK

**Keywords:** alumina, silica, cancer, biosensors, drug-delivery, theranostics, substrates, nanoplatforms

## Abstract

In recent years, remarkable progress in nanomedicine has been achieved, leading to the development of several nanocarriers which aim to enhance the therapeutic efficacy in cancer treatment. Owing to their high versatility and highly tunable physicochemical properties, alumina (Al_2_O_3_) and silica (SiO_2_) substrates represent promising and innovative nanoplatforms that are widely used in biomedical applications, such as drug-delivery, diagnosis, and biosensing in cancer. In particular, such platforms possess multiple advantageous properties, including mechanical stability, high loading capacity, tunable porosity, excellent biocompatibility, and in vitro and in vivo low toxicity. In this review article, we discuss their emerging role as biosensing platforms and drug delivery systems in oncology. As such, we describe how these substrates enable the incorporation of antibodies against various cancer biomarkers [e.g., cancer antigen 15-3 (CA15-3), serum amyloid A1 (SAA1), epithelial cell adhesion molecule (EpCAM), or human epidermal growth factor receptor 2 (HER2)] for the detection of multiple malignancies. Furthermore, we highlight the development of highly promising alumina- and silica-based platforms for drug delivery (e.g., chemotherapeutics, photosensitizers, or gene delivery agents) in cancer. Ultimately, by providing a comprehensive overview alongside a critical analysis, we demonstrate that such nanostructures represent promising platforms for potential clinical translation in cancer medicine, helping to mitigate the limitations of conventional cancer therapies.

## 1. Introduction

Biomedical applications are traditionally divided into diagnostic approaches, focusing on disease detection, and therapeutic approaches, which aim to treat the disease [1]. However a new approach, termed theranostics, has gained considerable attention [2,3]. This term was originally used by Funkhouser to describe a system that integrates both diagnostic imaging agents and therapeutic compounds, allowing simultaneous diagnosis and treatment [2,3]. This approach can also offer real-time feedback and allow treatment strategies to be tailored to individual patients, a property of great significance, especially in oncology given the heterogeneity of tumor evolution [2,3].

Cancer remains a predominant cause of morbidity and mortality worldwide [4]. Cancer cells exhibit a range of distinct biological hallmarks, including sustained proliferative signaling, resistance to programmed cell death, and the ability to invade surrounding tissues and metastasize [5]. In this context, biosensors have emerged as the means to measure a wide spectrum of tumor analytes and as a fast and accurate tool for cancer detection and monitoring [6]. Therefore, their use in early and accurate diagnosis allows for improved prognosis and application of effective treatment strategies, hence, reducing cancer mortality rate [6]. The current frontline of therapeutic approaches includes, among others, surgery, chemotherapy, radiotherapy, hormonal therapy, and the evolving field of immunotherapy [7]. Specifically, surgery and radiotherapy primarily eradicate localized tumors, whereas chemotherapy and hormone-based therapies are employed to control systemic disease by disrupting cell-division processes or growth signaling pathways [7]. 

However, in many cases, conventional antitumor strategies are often insufficient for effectively combating cancer [8]. Over the last two decades, the field of cancer nanomedicine has evolved significantly, driven by the immense need to overcome crucial challenges of cancer diagnostics and [8]. Nanotechnology seems to overcome, to some extent, several limitations of chemotherapeutics, including low tumor specificity, and therefore, unwanted and severe off-target effects that compromise the continuation and efficacy of the treatment [8]. The increased interest in improving and using nanomaterials for cancer is based on their capacity to deliver anticancer drugs and monitor disease progression and treatment response in real-time, while it minimizes the multiple life-threatening side effects [8,9]. 

Despite the fact that several previously fatal cancer types have been transformed into chronic and manageable diseases, core hindrances such as systemic toxicity, drug resistance, and tumor heterogeneity remain persistent challenges for modern medicine [7]. These limitations have led to the demand for the development of advanced, targeted and controllable drug delivery and diagnostic platforms, such as the nanomaterial-based systems [7,8,9]. Intriguingly, inorganic nanostructured materials, such as alumina (Al_2_O_3_) and silica (SiO_2_) substrates, have gained prominence as promising therapeutic platforms [10,11]. Highly investigated and tested systems, such as polymeric nanoparticles and organic nanocarriers, are currently facing a plethora of challenges, including low biological compatibility and stability, and increased in vivo toxicity [12,13]. Other nanocarrier platforms, such as metal–organic frameworks (MOFs) and calcium carbonate-based (CaCO_3_) carriers, encounter similar difficulties related to their stability in physiological conditions and potential toxicity, caused by the release of metal ions or organic linkers [14,15]. In contrast to the aforementioned substrates, alumina- and silica-based nanocarriers possess multiple advantageous properties, making them among the most promising candidate carriers for clinical theranostic purposes [10,11]. This is mainly attributed to the fact that they exhibit superior mechanical stability, tunable size, porosity, surface chemistry, increased surface-to-volume ratio, high biocompatibility, and resistance to uncontrolled drug release and degradation [10,11]. By exploiting these properties in combination with activating stimuli present in the tumor microenvironment, such as acidic pH, reactive oxygen species (ROS)-response mechanisms and overexpressed enzymes, on site dissolution of the nanomaterial-drug complex and subsequent localized drug release can be achieved [6]. An additional way to achieve on demand activation of drug administration is possible when applying light or ultrasounds [10,11]. Hence, these two substrates serve as ideal scaffolds for biosensing applications and chemotherapeutic or other anticancer drugs loading, providing a promising bridge between traditional cancer care and next-generation nanomedicine [10,11]. 

In this review, we present a comprehensive overview of alumina- and silica-based substrates, detailing their synthesis strategies, their unique traits and physicochemical properties, as well as their restricted limitations, both in functionality and efficacy. We further highlight their emerging role as potential biosensing platforms for cancer diagnosis and as drug delivery systems, engineered to mitigate the limitations of conventional cancer therapies. Lastly, we conclude that toward future clinical translation, it is crucial to consider the immunological interactions and biodistribution of alumina- and silica-based substrates.

## 2. Insights into Alumina and Silica Substrates

### 2.1. Synthesis and Design of Alumina Substrates

Alumina substrates, composed of aluminum oxide (Al_2_O_3_), have emerged in the field of nanomedicine due to their strength, heat conductivity, and excellent electrical insulation. In their standard configuration, alumina substrates acquire the form of flat ceramic plates that serve as foundational platforms for electronic circuits and microdevices. However, the anodization of aluminum promotes the transformation of its smooth surface into another form. Particularly, nanoporous anodic alumina (NAA) features a highly ordered honeycomb arrangement of cylindrical pores, vertically aligned and tightly packed. NAA exhibits high surface area, tunable surface chemistry, and optical responsiveness qualities, which have attracted significant attention in biomedical applications, particularly in biosensing and cancer theranostics [16,17].

**Figure 1:**
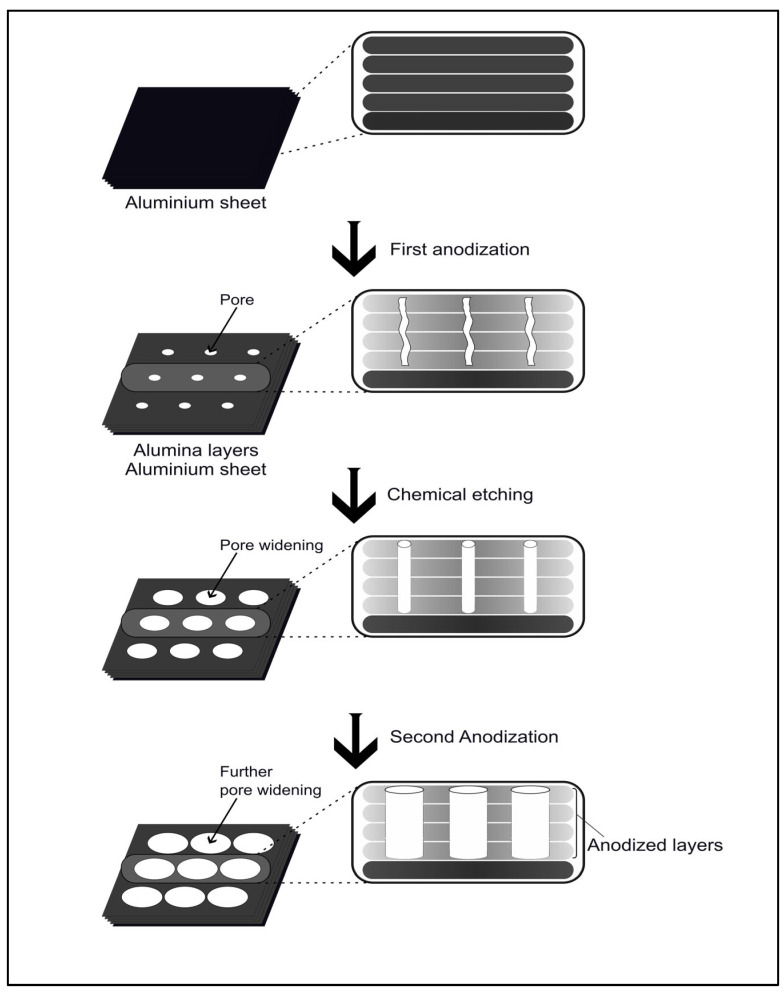
Two-step anodization. Schematic illustration of the two-step anodization process, consisting of two sequential electrochemical steps that yield highly ordered and functional anodic oxide layers. During the first step (**upper panel**), the aluminum film oxidizes and forms a poorly ordered porous alumina layer. Through chemical etching, the underlying surface forms a more ordered pore structure. Finally, the second anodization step (**lower panel**) proceeds, enabling controlled pore widening.

NAA is typically fabricated by electrochemical anodization of pure aluminum, using sulfuric, oxalic, or phosphoric acid electrolytes. Its pore pattern is formed under constant voltage, which promotes the oxide formation and dissolution, creating a self-organized porous layer [16]. Masuda and Fukuda introduced a method, known as two-step anodization, which improves the regularity of the pores (Figure 1). Particularly, in this process, the initial oxide is formed, removed, and used as a mold for the aluminum to maximize the uniform pore during the second anodization [18]. Of note, there are several anodization processes that are characterized by distinct variations. While mild anodization promotes precise control over oxide layer formation with slow growth kinetics, hard anodization facilitates the oxide development and results in the formation of deeper, well-defined pores [16]. Alternative processing methods such as voltage pulsing, annealing, barrier-layer thinning, funnel-shaped pores, and hierarchical structures endorse tune pore depth, geometry, and optical characteristics.

### 2.2. Synthesis and Design of Silica Substrates

Silica substrates are materials composed of silicon dioxide (SiO_2_), which may exist in either crystalline or amorphous forms and serve as versatile platforms across numerous biomedical and technological applications. A wide range of synthetic techniques are employed to generate mesoporous silica substrates, including sol–gel methods, microwave-assisted synthesis, and chemical etching. Among these, the sol–gel method remains the most valuable one, due to its low-temperature processing, tunable parameters, and facile functionalization [19]. In the aforementioned method, silica precursors such as silicon alkoxides undergo hydrolysis to form silanol groups (Si-OH), which subsequently create siloxane (Si–O–Si) networks. To obtain ordered mesoporous silica, structure-directing agents (SDAs), such as surfactants or block copolymers, are required to template periodic pore architectures, revealed upon template removal by calcination or solvent extraction. Furthermore, thin films can be fabricated through sol–gel-derived approaches, including evaporation-induced self-assembly (EISA), electro-assisted self-assembly (EASA), or Stöber solution growth method. While EISA typically produces horizontal pores and Stöber requires smooth and reactive substrates, the more recent EASA method enables vertically aligned channels on a variety of conductive surfaces. The latter is a key feature for enhancing mass transport in applications including biosensing and drug delivery [20].

### 2.3. Functionalization of Alumina Substrates

**Figure 2:**
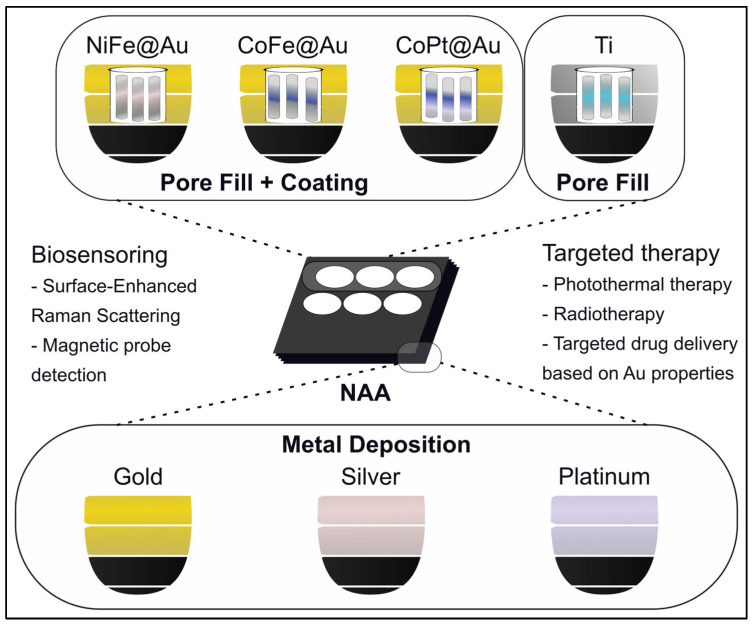
Metal deposition on Nanoporous Anodic Alumina. The nanoplatforms acquire new traits after functionalization via metal deposition, based on the coating material. Furthermore, the combination of these metals, either as coating or as payloads, gives the nanoplatforms higher specificity and application versatility.

The surface functionalization of alumina substrates is an essential step for their application in the field of cancer theranostics. One of the most-used functionalization methods of these substrates is metal deposition (Figure 2), which is achieved using techniques such as sputtering, evaporation, or chemical vapor deposition (CVD), effectively improving its conductivity, catalytic activity, or antimicrobial behavior. Another effective functionalization strategy involves coating alumina substrates with diverse thin films. In cancer theranostics, coatings based on metals such as gold, silver, platinum, palladium, titanium, nickel, and cobalt have been widely explored. In fact, silver-modified alumina substrates have been shown to rejuvenate fibroblast cells, underscoring the potential application of the aforementioned pads in the field of tissue regeneration [16,21]. An additional functionalization method is related to the formation of polymers like polypyrrole (PPy), that can be electropolymerized directly inside the pores, creating nanowires which change surface topography and cell adhesion behavior [22]. A distinct polymer that is used in the procedures mentioned above is polymethylmethacrylate (PMMA). Particularly, alumina coatings of PMMA, fabricated through the electrophoretic deposition (EPD) procedure, have shown increased alkaline phosphatase (ALP) enzymatic activity and strong osteoconductive potential. The latter represents a key characteristic that contributes to the translational relevance of findings in bone cancer and implant-related research [22]. Other chemical functionalization methods like silanization with (3-Aminopropyl) triethoxysilane (APTES), phosphonic acid grafting, atomic layer deposition (ALD) coatings, or sol–gel modification enable alumina surfaces to selectively bind to drugs, peptides, DNA strands, nanoparticles, or antibodies (Table 1). These methods are capable of turning these platforms into drug reservoirs, biosensors, or selective microfluidic interfaces [16]. Ultimately, alumina can be combined with polymers through EPD, modified with sodium cholate NaCh, forming hybrid films on steel or alumina surfaces [23].

### 2.4. Functionalization of Silica Substrates

**Figure 3:**
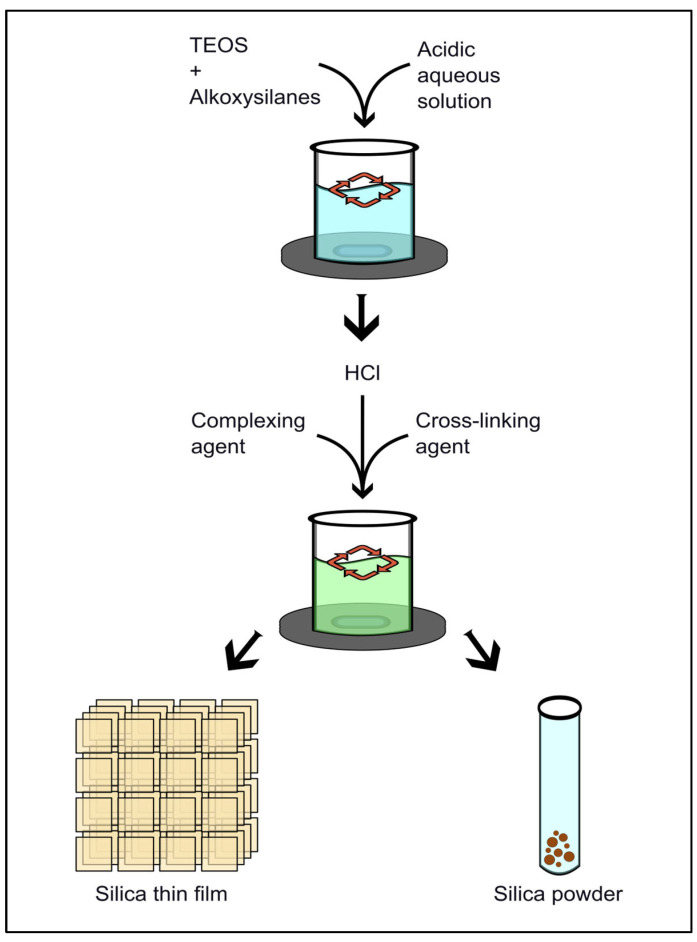
Sol–gel synthesis. Schematic illustration of a wet-chemical method leading to the formation of silica powders and thin films. Initially, TEOS, other alkoxysilanes, and acidic aqueous solution undergo hydrolysis (**upper panel**) and condensation reactions, forming a colloidal “sol” (**middle panel**). Upon addition of complexing and cross-linking agents, and by fine-tuning of pH, the sol evolves into a gel network with tunable particle size, porosity, and structural organization (**lower panel**).

Functionalization of silica substrates involves enriching the mesoporous surface with organic or inorganic groups, biomolecules, or ions in order to enhance hydrophilicity, stability, and selectivity [20]. This can be achieved via co-condensation during sol–gel synthesis (Table 1, Figure 3), as mentioned before, which distributes functional groups throughout the material. Alternatively, the post-grafting process is utilized to attach the groups following pore formation [24]. Representative functional groups, frequently employed in silica surface modification, include amino groups (e.g., APTES), which enable peptide conjugation and increase hydrophobicity; thiol groups (e.g., mercaptopropyl) for disulfide-based biofunctionalization; as well as carboxyl and hydroxyl groups for the adsorption of biomolecules [20]. Lastly, functionalization with PEG or other stabilizing groups mitigates silica hydrolysis in biological environments, thereby enhancing controlled delivery [20]. These groups also improve biocompatibility, since the release of solubilized silica and encapsulated drugs during dissolution may pose cytotoxicity risks [25].

### 2.5. Properties of Alumina Substrates

The biomedical appeal of alumina stems not solely from its exceptional structural robustness but also from its remarkable physicochemical adaptability, which enables diverse functionalization and broad applicability across medical contexts. In particular, alumina exhibits strong resistance to thermal and chemical degradation, coupled with increased mechanical durability. Notably, its chemical structure is often described as “onion-like” due to its multiple layers. Although there is ongoing debate regarding the number of distinct layers of the structure (two, three, or four), there is consensus that the outermost regions are enriched with electrolyte-derived species, while the inner domains consist predominantly of pure Al_2_O_3_. Lastly, the abundance of hydroxyl groups on its surface is particularly useful for further modification [16].

In the field of cancer research, alumina has been explored in three major directions: studying cancer cell behavior, building scaffold-like platforms for tumor microenvironments, and sensing biomarkers for diagnostics. Nanoporous alumina, especially when combined with PPy nanowires, enhances cell spreading, cytoskeletal extension, and motility in MCF and HeLa cells, which makes it useful for studies on metastasis or mechanical signaling [22]. Regarding bone-related cancers, polymethyl methacrylate (PMMA) alumina composite coatings have demonstrated enhanced metabolism in Saos-2 osteosarcoma cells and increased ALP activity, showing promising results in tumor-supporting scaffolds and bone–implant interfaces [23].

Perhaps the most active research area is biomarker detection using NAA-based optical biosensors. Structures such as Fabry–Pérot interferometers, rugate filters, or Bragg reflectors exploit optical interference effects, enhanced by pore geometry, to detect microRNAs, circulating tumor DNA, and protein biomarkers with increased sensitivity, rendering them suitable for liquid biopsy applications and early cancer diagnostics [16].

Altogether, alumina substrates ranging from dense ceramic plates to finely engineered nanoporous structures offer a highly adaptable material platform. Their ability to combine mechanical durability, surface tunability, optical properties, and biological compatibility position them as ideal candidates for integration into next-generation cancer-focused diagnostic and therapeutic systems.

To rationalize how alumina substrates interact with biological systems, it is important to consider the mechanistic role of pore architecture and surface chemistry. In the first case, in vitro and in vivo studies have demonstrated that nanoporous alumina membranes with 200 nm pores induce a stronger inflammatory response, characterized by increased macrophage activation and secretion of proinflammatory cytokines, compared to 20 nm pores [26]. Many additional studies support the differential behavior of cells grown on alumina substrates with deep versus shallow alumina pores, highlighting pore depth as a key mechanistic factor in regulating biological responses [27]. This represents a crucial design parameter for alumina-based biosensing and drug-delivery systems. Additionally, biological responses are strongly governed by surface chemistry in alumina-based substrates, including adsorption of biomolecules at the alumina surface. The physicochemical and mechanical properties of alumina substrates, such as hydroxylation and charge, significantly influence protein interactions and cellular behavior, modulating cell adhesion and morphology [28].

### 2.6. Properties of Silica Substrates

Mesoporous silica materials, defined by their interconnected 2–50 nm pore networks, exhibit high surface area (700–1000 m^2^/g) and large pore volume (>0.9 cm^3^/g) along with tunable pore size. The latter renders them appropriate substrates for efficient molecule loading, diffusion, as well as controlled drug release. Of note, their surface silanol groups improve their biocompatibility, thereby facilitating their chemical functionalization, resulting in enhanced material targeting and stability [19]. Particularly, ordered mesoporous thin films improve diffusion kinetics compared to disordered structures. Thus, they demonstrate increased suitability for biosensing applications [20].

Similar to alumina nanoparticles, the surface chemistry and pore architecture of silica-based substrates are important determinants governing biological responses. Surface chemistry critically influences biological responses, as variations in surface silanol density, charge, and chemical modification directly regulate interactions with cell membranes and intracellular processing. In parallel, mesoporosity and pore architecture modulate the effective contact area between particles and cells, as well as internalization processes [29]. Collectively, these parameters enable the rational design of biocompatible silica-based platforms, supporting their effective utilization in biosensing and drug-delivery applications [29]. For instance, in a recent study, Hasany et al. designed silica nanoparticles and demonstrated that, even in the absence of therapeutic or sensing moieties, surface chemistry alone can significantly influence cellular responses. Particularly, they developed silica nanoparticles bearing various surface functionalities, inducing differential cell viability in both 2D and 3D cellular systems [30]. Additional studies in RAW 264.7 murine macrophages demonstrate that the highly positive charged silica nanoparticles exhibit the greatest cell-surface adsorption and trigger higher cytotoxicity compared to other types. More broadly, nanoparticle surface chemistry has been shown to influence protein corona composition and inflammatory responses, a topic discussed later in this review [31]. In this context, protein corona formation relies significantly on the surface charge and physicochemical properties such as particle size, hydrophobicity, and chemical structure [31].

## 3. Theranostic Applications of Alumina and Silica Substrates

### 3.1. Beneficial Traits of Alumina Substrates for Biosensing Applications

**Figure 4:**
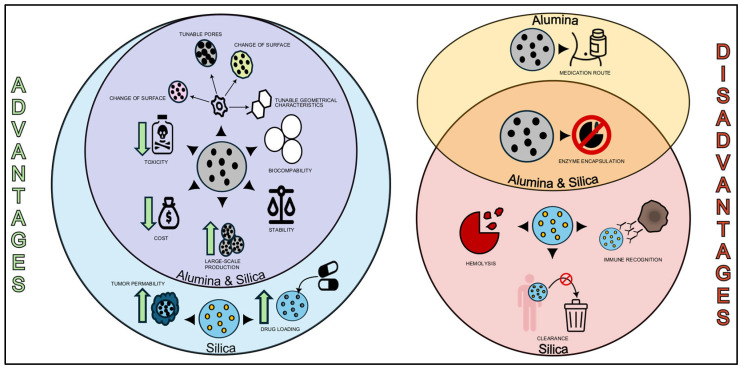
Advantages and disadvantages of alumina and silica substrates in cancer theranostics. A schematic overview of alumina and silica, comparing their key material properties, functional capabilities, and biomedical performance. The (**left panel**) highlights significant advantages such as tunable pore size and geometrical characteristics, surface functionalisation flexibility, high drug-loading capacity, increased tumor permeability, biocompatibility, stability, cost-effectiveness, low toxicity, and large-scale production feasibility. The (**right panel**) summarises the main limitations, which include haemolysis, immune recognition and clearance, enzyme encapsulation challenges, restricted circulation movement, and preferred medication routes. The Venn-diagram layout emphasises overlapping and complementary features across alumina and silica substrates.

As mentioned above, NAAs, due to their unique properties, are applied in a plethora of scientific fields, including biosensing. More specifically, NAAs have ideal characteristics for in vivo settings as they evade immune clearance, they are bioresorbable, and demonstrate high biocompatibility [32]. In line with this notion, alumina substrates, especially NAAs, present robust advantages for biosensing due to their high surface area, their tunable pore structures, enhanced chemical/physical stability, cost-effective fabrication, and high compatibility with various detection methods [17], as mentioned above. All the functionalization methods take advantage of the high density of hydroxyl groups reported on the aluminium oxide because of its preparation process, which can be further increased by boiling samples in oxygen peroxide. Alterations in the physical properties of the NAAs surface, such as optical response, electrode potential, and transmembrane potential, form the basis for the development of highly sensitive systems to the formation of the desired immunocomplex. Notably, the oligonucleotide-based sensors present high selectivity, specificity and affinity, even higher than those of antibodies [17]. All in all, these characteristics can be effectively used to modify the sensitivity of biosensors based on nanochannel blockage by designing nanochannels with suitable geometrical features considering the size of the biomolecules involved (Figure 4, Table 2). 

Of note, there are also limitations in the use of alumina substrates as biosensing platforms. As for the immunosensors, the principal challenges of non-labelled sensors regard their requirements in performing sufficiently high selectivity and physical properties changes. On the other hand, oligonucleotide-based sensors seem to overcome the limitations of immunosensors since they can be reproduced with increased accuracy, modified easily, while at the same time, large-scale production is possible, and their diversity is very high in comparison to antibodies. Other NAA biosensors, like the ones that incorporate enzymes, exhibit serious disadvantages, since enzymes’ structure is extremely sensitive, which complicates their use as sensors with high sensitivity and stability [17]. More specifically, the main challenges for enzymes in inorganic membranes refer to preventing denaturation function, from harsh surface interactions, ensuring efficient activity, encapsulation difficulties, managing enzyme orientation, and alleviating issues such as enzyme leaching or aggregation, often requiring specific immobilization techniquesto balance cost, stability, and activity for applications like biosensors or biocatalysis. More specifically, inorganic surfaces can provoke the unfolding of enzymes or stretch, altering the active site shape and reducing activity. The process of immobilizing an enzyme to a solid inorganic surface, particularly through strong covalent bonds, can inadvertently pull the protein into structural changes, leading to reduced catalytic activity or complete denaturation. All the aforementioned are further influenced by the dimensional characteristics of the platform. Moreover, enzymes can be encapsulated within solid structures, either by incorporating them in the manufacturing process or by filtering an enzyme solution through a membrane so that the enzymes will be trapped in the pores. Moreover, with the layer-by-layer method, enzymes can be trapped in a network of polyelectrolytes of alternating charges. Although entrapment and encapsulation offer a shielding microenvironment within the solid substrates and are characterized by the minimal effect on the enzyme structure, the catalytic efficiency can be hampered by mass transport limitations. Finally, as long as it concerns the stimulus complexity in enzyme based biosensors, the pH levels in TME can denature enzymes before they are even fully immobilized [33].

### 3.2. Biosensing Applications of Alumina Substrates for Cancer Detection

Biosensors are widely used analytical tools in healthcare, enabling early diagnosis as well as real-time monitoring of diseases and treatments. These capabilities stem from their high specificity and sensitivity, combined with their cost-effective nature and scalability [34,35,36]. Biosensors consist of a bio-receptor, which recognizes the target molecule, and a transducer, which converts this interaction into a measurable signal [34,35,36]. Their classification is commonly based on: (i) the type of molecule that binds to the bio-receptor and (ii) the type of signal transduction employed [34,35,36].

**Figure 5:**
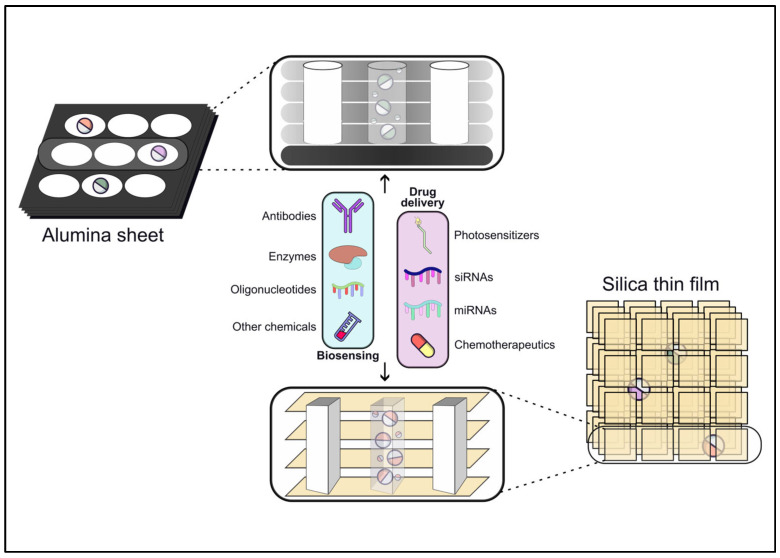
Alumina- and silica-based agents for biosensing and drug delivery. Schematic representation showing the surface modifications of alumina and silica substrates for biosensing applications (left panel), along with the various drug-loading carriers that can be incorporated into these materials, that renders them appropriate for drug-delivery systems (right panel). A wide range of functional agents, including antibodies, enzymes, oligonucleotides, photosensitizers, siRNAs, miRNAs and chemotherapeutics, can be immobilized or encapsulated within the nanoplatform (middle panel). The scaffolds that are designed (right panel) enable selective detection of various cancer biomarkers, targeted therapy and multifunctional theranostic applications.

Alumina substrates, due to their unique properties, are broadly applied in a plethora of scientific fields, including biosensing (Figure 5). NAA-based biosensor designs have been developed for the diagnosis of various diseases [37]. In this section, we focus on the NAA-based sensors used for cancer diagnosis and disease progression monitoring that have been developed over the past 15 years.

An important study in 2011 introduced a method designed for the detection of cancer antigen 15-3 (CA15-3), a biomarker associated with breast cancer progression. Porous anodic alumina was used as the substrate, and antibodies specific to CA15-3 were immobilized on the surface of its pores. Following the binding of the biomarker, gold nanoparticles (AuNPs) were added as blocking agents. This resulted in partial blocking of the passage of electroactive elements, preventing them from reaching the electrode located at the bottom of the pores. The reduction led to decreased oxidation, thus resulting in a decrease in the measured signal, meaning the detection of CA15-3 [38]. Another recent development involves a system for detecting circulating tumor cells (CTCs), which serve a key role in metastasis. Particularly, biotinylated epithelial cell adhesion molecule antibodies (anti-EpCAM) were fixed on gold-coated nanoporous alumina. Upon formation of antibody-cell complexes, a shift occurred in the interferometric reflectance spectrum, enabling detection of the captured CTCs [39]. Furthermore, Jae-Sung Lee et al. developed a biosensor for the detection of lung cancer, taking advantage of the lung cancer-specific biomarker serum amyloid A1 (SAA1). In greater detail, they coated an NAA substrate with Ni/Au and restrained SAA1-specific antibodies. Using both localize surface plasmon resonance (LRSP) and interferometry, the researchers achieved a limit of detection (LOD) of 100 ag/mL, highlighting the increased sensitivity of that method [40]. Another application using an immuno-based biosensor was reported in a recent study, in which anti-CD3 and anti-EpCAM antibodies were fixed on the surface of porous alumina to efficiently capture exosomes, as cancer patients typically exhibit increased exosome levels compared to healthy individuals. The captured exosomes blocked the entrance of the pores and reduced ion flow (measured using a picoammeter) [41]. Moreover, a recent study reported the construction of an enzyme-based sensor for the detection of lung, colorectal and parotic cancer biomarkers using cytochrome C. Particularly, they immobilized trypsin to an NAA platform in order to cleave the heme groups of cytochrome C and produce peptides that can oxidaze 2,2-azino-bis(3-ethylbenzothiazoline-6-sulfonic acid) (ABTS) to create ABTS-anions. In the presence of hydrogen peroxide (H2O2), the solution becomes green, indicating high risk for cancer progression [42]. 

A distinct category of biosensors includes oligonucleotide-based sensors, which have been developed for the detection of breast, colorectal and cervical cancer [43,44,45]. In the first case, researchers aimed to detect miR-99a-5p, which is an early indicator of breast cancer. The NAA pores were filled with rhodamine B and capped with a complementary oligonucleotide connected to (3-Aminopropyl) triethoxysilane (APTES). When the miRNA binds to the oligonucleotide, the cap is removed, the fluorogen is released and the absorption is measured [45]. In line with this notion, for the detection of changes in the levels of the oncomarker 8-oxo-7,8-dihydro-2-deoxyguanosine (8-oxo-dG), a sequence of DNA with a tertiary structure that can bind a molecular target, known as an aptamer, was used as a cap [44]. A supplementary strategy for rhodamine implementation B system has been published by Hernández-Montoto et al., who synthesized oligonucleotide probes in order to detect hr-HPV types which increase the risk of cervical cancer [43]. Additionally, a research team recently reported the synthesis of an alumina nanoporous substrate coated with Au that employs an aptamer in order to bind cancer biomarker Mucin-1, thus constricting the passage of ions through the pores and creating a detectable signal [46]. Similarly, AuNPs-engineered alumina nanoporous membranes have been developed, aiming to achieve sensitive electrochemical detection of exosomes, while a nanochannel based on dendrimer-gold network and aluminum oxide arrays enabled the detection of miRNA-122, highly associated with liver cancer [47,48]. 

### 3.3. Beneficial Traits of Silica Substrates for Biosensing Applications

Silica-based systems and mesoporous silica nanoparticles (MSNs) provide several unique traits that bring robust benefits for developing biosensors. One of their most useful characteristics refers to the stable functionalization of their surface. Thus, various functional groups such as thiol, amine, hydroxyl, halogen, and others are attached to the silica surface in a one-step reaction with different alkoxysilanes. This feature enables the use of versatile functionalization strategies, facilitating the desired final functionalization on the NP surface. The tunable porosity of MSNs is another distinctive property that renders their use suitable for fabricating biosensors. Their pores can be filled with signaling molecules such as dyes or electroactive species for developing optical or electrochemical biosensors, respectively. Furthermore, the distribution of their molecules can be modified by surface-functionalization and pore-blocking species. Targeted release of cargo molecules can be attained by attaching pore-blocking species to the MSNs via stimuli-cleavable linkers. Therefore, the structure of the employed linkers encompasses functional groups that can be cleaved upon reaction with specific reagents (e.g., disulfide groups for cleavage by reduction agents, glutathione), change in pH (e.g., hydrazone or acetal linkages for cleavage by acidification), or after the interaction with other external factors such as magnetic field or light irradiation. Another unique trait of MSNs refers to the fact that they are not optically active and conductive without the loaded signaling molecules, which results in the enhancement of the signal/noise ratio [49].

The fabrication of biosensors for cancer biomarkers based on MSNs is also promising since their large-scale production is affordable. However, when expensive recognition elements such as antibodies or aptamers are used, the cost of the final products is elevated. Nevertheless, heterogeneous post-modification may enable the redeployment of residual recognition elements following centrifugation of the MSNs, thereby reducing the overall cost of biosensor production [49]. 

It is obvious that the silica-based systems offer unique advantages in drug delivery and biosensing. Despite their robust potential in the aforementioned diagnostic applications for cancer, a vital challenge lies in their transport (diffusion, convection) through different media (circulation, membranes, porous materials) to ensure that the biosensor reaches the desired surface or reaction site with high specificity and affinity [50]. For example, the mass transport of molecularly imprinted polymers (MIP) films incorporating silica is impeded from slow diffusion to cavities with reduced accessibility, thus restricting the selectivity of MIP-assisted biosensing applications. As long as it concerns the enzyme-based biosensors, a significant limitation is related to the enzyme encapsulation capacity of the porous materials. 

Although numerous efforts have been made to develop MSNs for biosensing applications, their performance is still impeded by several significant limitations. Specifically, a key challenge is assessing and mitigating their biocompatibility and toxicity in vivo, since surface silanol groups may interact with red blood cells and induce hemolysis or immune reaction [51,52]. These traits depend on their properties and specifically on their particle size, dose, surface chemistry, and the presence of any drug payload. Surface functionalization of MSNs, particularly through the application of biocompatible polymer coatings decrease cytotoxicity by decreasing particle aggregation while improving cellular uptake efficiency and reducing recognition by the immune system. Furthermore, another way to diminish the toxicity of the carriers refers to the coating of MSNs with biocompatible polymers such as 3-APTES or PEG, which decrease interactions with cell membranes and thus mitigate their potential to induce toxicity. Optimizing the size of NPs is also significant as the NPs that are too small are possible to accumulate in organs such as the kidneys, whereas oversized particles risk causing embolism in blood vessels or triggering immune responses. Moreover, as long as it concerns the specificity and sensitivity of MSNs, the surface chemistry and the particle size are two critical parameters to balance, which are correlated by the structural properties of the materials. For instance, expanding pore size promotes the selective accommodation of larger-sized analytes, while reducing surface area and deteriorating detection sensitivity. Thus, design compromise is required to attain enhanced sensitivity and specificity in detection, especially for complex clinical use [53] (Table 2). 

### 3.4. Biosensing Applications of Silica Substrates for Cancer Detection

Determining the presence and measuring the concentration of cancer-related biomarkers in body fluids enables both the detection of cancer across various stages and the tracking of treatment effectiveness [54]. Nanobiosensors, which involve mesoporous silica nanoparticles (MSNs) and mesoporous silica films (MSFs), are useful tools for the incorporation of sensing elements and the subsequent detection of those biomarkers in the diagnosis of cancer [49,55] (Figure 5). Several MSN-based biosensors have been developed to target biomarkers of different cancer types [49].

In breast cancer, electrochemiluminescence (ECL)-based cyto-immunosensing strategies are widely used for the detection of various biomarkers. Particularly, a recently described method utilizes a vertically oriented silica-based mesoporous material (SBMM), which is integrated with an electrode and combined with a chitosan–luminol composite. Chitosan serves as an adhesive matrix to enhance stability and facilitate antibody immbilization for the selective detection of metastatic breast cancer cells [56]. Furthermore, another ECL immunosensor for breast cancer has been reported for the quantification of the biomarker CA15-3. In this system, Ru(dcbpy)_3_^2+^, PEI, and AuNPs are immobilized on dendritic mesoporous silica nanoparticles (DMSNs) to form an efficient ECL sensing platform. The specific antigen–antibody recognition of CA15-3 introduces Cu_2_O@poly(dopamine) nanoparticles as dual quenchers, resulting in a concentration-dependent decrease in the ECL signal [57]. Of note, a recent scientific work described the synthesis of a peroxidase-mimicking mesoporous silica–gold nanocluster hybrid platform (MSN–AuNC–anti-HER2), where human epidermal growth factor receptor 2 antibodies (anti-HER2) are immobilized on the MSN surface, and gold nanoclusters are confined within the mesopores. This platform catalyzes H_2_O_2_-mediated oxidation of TMB for colorimetric detection of HER2-positive breast cancer cells [58]. In line with these findings, Yu Chen et al. fabricated a mesoporous silica-based ECL system modified with phenyl-boronic acid, loaded with Ru(dcbpy)3^2+^, and capped with polyhydroxy-functionalized AuNPs, allowing breast cancer cell detection through signal modulation. Specifically, this system exploits endogenous H_2_O_2,_ produced by MCF-7 cells, which oxidizes the arylboronic ester linker, triggering the release of Ru(phen)3^2+^ and subsequent enhancement of the ECL signal [59].

Numerous studies have employed silica-based nanoplatforms as carriers and signal amplification scaffolds for the detection of lung cancer biomarkers. For instance, Yuan Zhang et al. fabricated a core-shell nanozyme (CPT/DM-FA) based on dendritic mesoporous silica nanoparticles with a MnO_2_ shell, which enables triple-mode glutathione (GSH) sensing through MnO_2_-mediated signal transduction (fluorescence, UV–vis, and colorimetric) and specific cancer cell detection (through folic acid surface modification) [60]. Another scientific work refers to the fabrication of a biosensor for cytokeratin fragment 19 (CYFRA 21-1) detection. The latter utilizes a pH stimulus-responsive release strategy wherein polyethylenimine-modified silica (SiO_2_-PEI) serves as a carrier, glucose functions as the inducer for controlled release, and the ECL signal is generated through the specific interaction between antibody and antigen [61]. Of note, an electrochemical immunosensor for CYFRA 21-1 detection has been recently developed. This employs a silicon nitride–molybdenum disulfide composite on multi-walled carbon nanotubes as the sensor platform, with core-shell magnetic mesoporous silica nanoparticles@gold nanoparticles acting as signal amplification elements. The antibodies that target CYFRA21-1 are immobilized on the platform via electrostatic/ionic interactions, enabling sandwich-type voltametric immunosensing for lung cancer biomarker detection [62]. In line with these findings, a scientific team recently described an immunosensor for the detection of CYFRA 21-1 in lung cancer based on a personal glucose meter. This immunosensor is fabricated by utilizing polyethylenimine-modified mesoporous silica nanoparticles (MSN-PEI) loaded with glucose and CYFRA 21-1 antibody-labeled gold nanoparticles. The antibody–antigen connection triggers glucose release, and its subsequent measurement indirectly reflects the presence of CYFRA 21-1 [63].

In prostate cancer, there are different nanoprobes used for biosensing. Particularly, a scientific team created fluorescent nanoprobes composed of N-acetyl-l-cysteine capped-copper nanoclusters (NAC-CuNCs) incorporated into three-dimensional mesoporous silica particles (MSiO_2_), combined with MnO_2_ nanosheets. The latter refers to a turn-off/turn-on fluorescent detection platform for prostate cancer biomarker acid phosphatase (ACP), where the fluorescence signal stems from MnO_2_ reduction. As a result, ACP-triggered hydrolysis of l-ascorbic acid-2-phosphate reduces MnO_2_ to restore fluorescence [64]. In another recent work, a state potentiometric sensor for sarcosine, a prostate cancer biomarker, was designed. This was fabricated by utilizing a molecularly imprinted polymer (MIP) polymerized over silica nanoparticles to achieve high selectivity in phosphate-buffered solution and simulated body fluid for prostate cancer detection [65]. Furthermore, a recent study is related to the synthesis of pyrroloquinoline quinone (PQQ)-decorated mesoporous silica nanoparticles (MSNs) functionalized with anti-prostate specific antigen (anti-PSA) antibody. This antibody act as a catalytic and recognition element in a sandwich-type colorimetric immunoassay, where PSA bridges magnetic capture beads and the nanoparticles to enable signal amplification through catalytic reduction of Fe(III)-ferrozine to Fe(II)-ferrozine [66].

In the context of pancreatic cancer diagnostics, a controlled-release electrochemical immunosensor targeting cancer antigen 19 (CA 19-9) has been developed, employing glucose-loaded mesoporous silica nanoparticles (MSNs) capped with ZnS and functionalized with anti-CA19-9 antibody. The system achieves release through dithiothreitol (DTT)-mediated disulfide bond cleavage, thereby liberating glucose for subsequent oxidation on a three-dimensional cactus-like nickel–cobalt layered double hydroxide/copper selenide nanosheet-modified electrode, where the electrochemical signal is generated [67]. In line with these findings, a multichannel light-addressable photoelectrochemical (PEC) sensor has been engineered to enable multiplexed detection of pancreatic cancer biomarkers, including glypican-1 (GPC1), carcinoembryonic antigen (CEA), and glutathione (GSH). This platform integrates gold nanocluster/graphene oxide-based PEC immunosensors for GPC1 and CEA with carbon dot@mesoporous silica bead-based PEC sensors for GSH, thereby facilitating simultaneous and highly sensitive biomarker analysis [68].

As for cervical cancer, MSNs are widely employed as platforms for the immobilization of sensing elements. Particularly, a scientific team developed MSNs loaded with methylene blue (MB) and capped with chitosan, which act as a redox probe nano-depot, functionalized with anti-E6 antibody 2 for HPV16 E6 oncoprotein detection. A glassy carbon electrode modified with dendritic palladium–boron–phosphorus nanospheres (PdBP-NSs) and anti-E6 antibody 1 enables sandwich-type electrochemical sensing, where MSNs modulate MB electropolymerization and amplify the signal response [69]. In line with these results, a recent work reported the synthesis of a sandwich-type electrochemical immunosensor for cervical cancer biomarker SCCA, which utilizes highly branched PtCo nanocrystals (PtCo BNCs) as electrode substrates for enhanced conductivity and antibody loading, combined with dendritic mesoporous SiO_2_@AuPt nanoparticles (DM-SiO_2_@AuPt NPs) loaded with thionine as an electroactive signal label for amplified electrochemical detection [70]. A label-free electrochemical cytosensor for HeLa cells employs folic acid-functionalized vertically ordered mesoporous silica films (VMSFs) on an indium tin oxide (ITO) electrode to prevent direct cell contact while maintaining electrode activity. The specific adhesion of HeLa cells to the surface impedes the transport of the Fe (CN)6^3−^ redox probe through the nanochannels, resulting in a decreased electrochemical response that is used as the analytical signal for quantitative determination [71].

In ovarian cancer, biphenyl-loaded mesoporous silica nanoparticles (BDMSNs) combined with multiplex lateral flow immunoassay (MLFIA) enable simultaneous detection of ovarian cancer biomarkers CA125 and Human Epididymis Protein 4 (HE4), leveraging the aggregation-induced emission property and high biotin–streptavidin affinity of BDMSNs as fluorescent signal reporters with robust antibody enrichment [72]. A sandwich-type magneto-immunosensor for simultaneous detection of three ovarian cancer biomarkers (HE4, Alpha-fetoprotein-AFP, and CA 125) employs screen-printed electrodes combined with electroactive nanomaterials, including gold nanoparticles, CdTe, and PbS quantum dots conjugated with specific antibodies, and MSNs, serving as signal amplification elements for enhanced electrochemical signal readout [73].

As regards other cancer types, an electrochemical cytosensor for colorectal cancer stem cell (HT-29 CSC) detection utilizes a nanocomposite of mesoporous silica nanoparticles (MSNs) and platinum nanoparticles (PtNPs) on a glassy carbon electrode with biotinylated monoclonal antibodies targeting the Cancer Stem Cell (CSC) marker CD133, enabling sensitive cell detection through enhanced mass and charge transfer [74]. A dual-signal-amplified sandwich-type electrochemical immunoassay for CEA detection employs dual-labeled mesoporous silica nanospheres (amine-functionalized SBA-15 with entrapped Au nanorods and covalently conjugated HRP and anti-CEA antibody) combined with NiO@Au- and anti-CEA antibody-decorated graphene as a conductive layer for remarkable sensitivity enhancement [75].

### 3.5. Beneficial Traits of Alumina-Based Drug Delivery Systems

Given that a wide range of nanomaterials have been developed to achieve targeted drug release, inorganic nanoporous structures such as NAA and mesoporous silica substrates have attracted increased interest from researchers due to several factors. More specifically, NAA demonstrates a series of advantages for the development of drug delivery systems due to its excellent physicochemical and biocompatible properties. In line with this notion, NAA not only present excellent chemical inertness, enhanced mechanical strength, tunable chemistry, and controlled pore dimensions, volumes for loading and releasing drugs in a controlled manner, but also a cost-effective fabrication process [76]. The geometrical characteristics of these materials, such as pore dimensions, size, shape, interpore distance, and length, can be adjusted by varying anodization voltage, type, electrolyte concentration, and temperature. Thus, the tunable features of NAA make it an appropriate material for the loading of different anti-cancer drugs with high affinity and increased drug loading efficiency. Regarding drug release from NAA platforms, it is primarily based on molecular diffusion from the pores and is mainly governed by the pore dimensions. Therefore, the adjustment of pore diameter and pore depth has been considered a common strategy to control drug release performance [77]. Conventionally anodized aluminum is capable of forming a self-organized nanochannel array, characterized by a precisely regulated pore size distribution, which secures the high specificity in delivering drugs in targeted cells [78]. All in all, the physicochemical properties, biocompatibility, stability, and customizable surface functionalization of NAA render them as a highly effective platform for both high-affinity drug loading and precise, targeted drug delivery (Figure 4, Table 2).

On the contrary, regarding the limitations of NAA for drug delivery platforms, these mainly arise from their restricted loading capacity, which refers to their inability to encapsulate large quantities of their payload or extended chemical compounds (unable to load large drugs). Furthermore, the inadequate cellular uptake of some agents reduces the therapeutic efficiency of the antitumor agent, which may result in their aggregation in healthy organs. This point reflects the limitations of certain drug delivery NAA-based systems in clinical use [79]. However, the aforementioned limitation can be easily reversed by taking advantage of stimuli-responsiveness of alumina. Leveraging internal or external stimuli, such as pH or temperature, enables site-specific drug release, thus preventing any interaction with the healthy tissues. Finally, alumina-based materials lack inherent stimulus-responsive activation, thus limiting their spatiotemporal control in complicated biological settings. However, hybrid designs, especially rare-earth-doped alumina systems, reduce their functional restrictions by including redox activity, antimicrobial effects, and age-resistant properties. The aforementioned techniques allow the conversion of alumina from a structural material to a multipurpose bioactive platform, rather than a trigger-dependent therapeutic system [80].

### 3.6. Drug Delivery Systems Based on Alumina Substrates for Cancer Therapy

Alumina substrates have significantly emerged within the field of nanomedicine as scaffolds for the development of highly promising platforms, particularly for drug delivery applications in cancer [76,79,81] (Figure 5). Their superiority over alternative materials arises from their unique combination of physical and chemical properties. Alumina surfaces can additionally be coated with biodegradable, chemical, or pH-responsive agents to regulate and trigger drug release [81]. Given that these substrates are characterized by increased biocompatibility, decreased toxicity, significant mechanical strength, and chemical resistance under both oxidizing and reducing conditions, the aforementioned features position them as an exceptional, targeted drug delivery system, extremely necessary to be exploited for therapeutic purposes in oncology [76,79,81].

Porta-i-Batalla et al. developed NAA structures with varying pore dimensions, aiming to investigate the relevance of pore architectures with drug release rates. In this research, doxorubicin (DOX), a chemotherapeutic agent highly efficient in treating various types of cancer, was used as a model drug [81,82]. Interestingly, they demonstrated that these fabricated NAA structures prevent the undesired high initial drug release burst, thus providing a more stable and sustained drug-release profile. This controlled behavior reduces the risk of high-dose drug exposure and enhances local therapeutic availability [81,83,84]. Overall, these findings underscore the potential of using DOX-loaded alumina structures to achieve targeted elimination of tumor cells combined with reduced systemic toxicity. Another scientific work supporting targeted delivery approaches based on nanomaterials was reported by Gao et al., describing the synthesis of hyaluronic acid-coated mesoporous hollow alumina nanoparticles (HMHAs), applied for the treatment of liver cancer [85]. Particularly, HMHAs were loaded with paclitaxel—another widely used chemotherapeutic drug—and then investigated for their efficacy in drug delivery. A comparative analysis that was held between the free drug and drug-loaded HMHAs, proved that the latter exhibited higher cellular uptake, therefore, increased delivery of paclitaxel in the liver tumor tissues, and significantly elevated apoptosis induction in cancer cells of the liver [85,86].

Of note, Ajalli et al. developed a more advanced drug transport system, the Chi-tosan/Gamma-Alumina/Fe_3_O_4_@5-FU nanocarrier, which induced cytotoxicity in breast cancer cells (MCF-7 cell line) [87]. Tested with various loading assays, these new nanocomposites achieved controlled, stable and pH-sensitive release of 5-FU, also known as the pyrimidine analog fluorouracil, at the acidic environment of the tumors. The results extracted from flow cytometry analysis and (3-(4,5-Dimethylthiazol-2-yl)-2,5-Diphenyltetrazolium Bromide) (MTT assay), delineated that these nanocarriers induce increased levels of DNA damage and finally, trigger apoptosis in breast cancer masses [87,88]. Following a similar design strategy, Nafaji et al. and Nematollahi et al. reported two distinct nanocarrier composites with similarly high performance. In the first case, curcumin—a natural polyphenolic phytoalexin used in cancer therapy—was delivered in lung cancer cells and L929 cell line (used to test the biocompatibility of the transport system), via the Sodium Alginate/Zein/γ-Alumina nanocarriers, providing a promising, highly biocompatible and tumor-targeting drug delivery platform [89,90]. More recently, the anticancer effect of curcumin was also demonstrated using SA/PVP/γ-Al2O3 nanocarriers and carboxymethyl starch-modified gamma alumina nanoparticles for targeted delivery in colon and breast cancer therapy, respectively [91,92]. In the second case, the drug model of the study was quercetin (QC), a natural flavonoid used as an anticancer agent, which was loaded in Chitosan (CS)/Polyvinylpyrrolidone (PVP)/γ-Alumina nanocomposites and tested on MCF-7 breast cancer cells [93,94]. The QC-loaded CS/PVP/γ-Alumina nanocarriers showed significant superiority in achieving targeted eradication of cancer cells compared to treatment using only free QC [93].

More recently, Jia and colleagues moved the field of nanomedicine a step further by developing a system that enhanced the efficacy of metalloimmunotherapy by combining it with conventional anti-cancer therapy [95]. Under this scope, they created a magnesium-containing and methotrexate/5′-C-phosphate-G-3′ (CpG)-loaded nano-aluminum carrier, called NanoAlum [95]. Peritumoral injection of NanoAlum effectively converted “cold” tumors into immunologically active or “hot” tumors, by enhancing intratumoral T cell infiltration and activity. More explicitly, these nanocarriers enhanced the antigen-specific, anti-tumor immunity mediated by T-cells, leading to the apoptosis of cancer cells [95,96]. Furthermore, a recent study from Sichuan University reported the fabrication of nanocarriers, used to combine immunotherapy and photo-thermal therapy (PTT) [97,98]. Hence, they proceeded in coating alumina (Al_2_O_3_) nanocarriers with polydopamine, a material that exhibits high photothermal efficiency [98]. The main purpose of the study was to eliminate cancer cells by triggering downstream immune responses that concurrently contribute to avoiding tumor relapses [98]. After testing the nanocarriers in vitro, in terms of cellular uptake and cytotoxicity, they investigated their efficacy in vivo by injecting them into B16F10 melanoma allografts in mice. In the next steps, the mice underwent irradiation, which shrinks the tumor masses, resulting in the release of tumor-associated antigens. Thus, they described a mechanism by which the tumor antigens are being presented on the surface of mature dendritic cells, leading to immune responses activation [98]. The combinatorial administration of Al_2_O_3_ nanocarriers together with the immune adjuvant CpG effectively reduced melanoma tumor volume and the impending risk of antigen-mediated metastasis or tumor recurrence [98,99].

### 3.7. Beneficial Traits of Silica-Based Drug Delivery Systems

Silica and porous silica materials can be used as versatile platforms for drug delivery, overcoming challenges such as poor drug solubility, limited biodistribution, and lack of selectivity. Their high drug-loading capacity, which arises from to their ability to encapsulate large amounts and release drugs in a controlled manner, has driven extensive research in this area. Their unique characteristics of high surface area, controllable pore structure, and increased biocompatibility render them as a highly efficient tool for drug delivery. More specifically, MSNs exhibit exceptionally high surface areas, reaching approximately 1000 m^2^/g and pore volumes in the range of 0.6–1.3 cm^3^/g, features that markedly improve their drug-loading capacity and overall delivery efficiency. The surface of MSNs can be easily modified with various functional groups, securing the specificity of drug delivery and the controlled release of cargo. Their size and shape can be tuned, ranging from 20 to 600 nm in diameter, and from spherical to rod-like morphologies. Furthermore, these nanoparticles can passively enter tumors through the enhanced permeability and retention effect, and they can also be actively targeted by various ligands, while its PEGylation prolongs circulation time and availability. Additionally, MSNs not only exhibit low toxicity in vivo, but also have been utilized for co-delivery of anticancer drugs and siRNA, offering a potential strategy to overcome multidrug resistance in chemotherapy. Overall, MSN represent a robust and useful tool for novel and reliable cancer therapies, with potential for combinatorial therapies and gene delivery in oncology (Figure 4, Table 2) [25].

Despite the numerous advantages of silica substrates as drug delivery systems, they also exhibit some key limitations. Firstly, silica-based nanoparticles are preferably injected intravenously, and then, they circulate in the blood stream until they ascertain their target. One fundamental obstacle of this medication route refers to the prevention of premature clearance from the systemic circulation and the reassurance of the safe and long-term application of nano-DDSs. The latter is also described as hemocompatibility which is the interaction of foreign material with the blood system. Another challenge for silica-based systems is related to the avoidance of hemolysis. The underlying mechanism for hemolysis of amorphous SNPs is not fully understood but there are some hypotheses with the most prevalent supporting that the amount of surface silanol groups are responsible for the hemolysis in both SNPs and MSNs. More specifically, the degree of hydrophilicity (related to surface silanols -SiOH) and hydrophobicity (related to surface siloxanes -Si-O-Si-) of silica particles is a suggested determinant of the aforementioned toxicity. There are distinct proposed mechanisms to reduce the toxicity of silica-based systems provoked by hemolysis. For instance, thermal treatment of silica dust alleviates the structure, decreases the population of surface silanols, and accomplishes a reduction of their hemolytic activity. Other chemical treatments severely affecting the surface and consequently decreasing the hemolytic action of silica based systems have been also described to the involvement of silanols such as the adsorption of polymers (e.g., poly(2-vinylpyridine-N-oxide) (PVPNO), proteins, and lipids like albumin, lecithin, serum, plasma and corona, chloroquine, and aluminum compounds (e.g., AlCl_3_, aluminosilicate compounds, and aluminum lactate). A reduction in hemolytic activity has also demonstrated following the substitution of surface hydroxyl groups with trimethylsilyl groups and through hydrofluoric-acid etching of silica particles. Moreover, particle size appears to influence hemolytic behavior in both micro- and nanoscale silicas since among micrometric particles, smaller quartz species exhibit greater membranolytic potential than their larger counterparts. Finally, the hemolytic response of silica particles is also modulated by environmental factors, including temperature, pH, and the physicochemical characteristics of the surrounding medium [100].

As long as it concerns the immune responses, one major factor that must be considered, in these treatments is the activation of the immune and complement system. The activation of the complement system is influenced not only by particle size, surface charge, and functional group density, but also by surface curvature, and especially, the presence of surface defects (Figure 4, Table 2) [101].

Additionally, pH-based triggers are limited in deep-seated cancers because intratumoral acidity is low, spatially varied, and not specific to cancer, resulting in reduced treatment consistency or off-target activation. Although light-based triggers provide temporal control, they present poor tissue penetration and require oxygen for photodynamic treatment (PDT). They also exhibit difficulty in restricting thermal or photochemical effects at depth without causing collateral damage. Hybrid systems that combine tumor-microenvironment cues, such as pH- or enzyme-enhanced uptake, with/or externally applied energies such as NIR light, ultrasound, magnetic fields, or ionizing radiation to improve both selectivity and depth reach, are vital for their development. Such multimodal designs transform weak or unreliable single triggers into robust, spatially and temporally controlled activation mechanisms suitable for deep solid tumors [102].

### 3.8. Drug Delivery Systems Based on Silica Substrates for Cancer Therapy

Silica substrates and mesoporous silica nanoparticles (MSNs) have emerged as efficient carriers for therapeutic agents against cancer cells [52]. This ability is attributed to a wide range of internal (pH, redox potential and enzyme activity) and external (light, temperature and magnetic field) stimuli that mediate the intracellular release of drugs targeting neoplastic cells [103]. In this section, we present a concise overview of silica-based materials employed as drug-delivery vehicles, which are capable of overcoming various limitations frequently encountered in conventional cancer therapies, such as poor distribution, multidrug resistance (MDR) and non-specific targeting, commonly observed in traditional cancer therapy [104] (Figure 5).

MSNs have been extensively investigated in cancer drug-delivery research, compared to silica substrates, which typically promote localized rather than systemic drug release [105]. Notably, Lebold et al. developed mesoporous thin silica films featuring nanometer-sized pores, which were utilized as drug carriers for DOX [106]. Of note, drug diffusion and dynamics inside the nanopores are regulated by pore size and surface modifications [106].

Additional studies demonstrate that DOX-loaded MSNs show encouraging outcomes, including in vivo inhibition of glioma growth, antitumor efficacy in breast and pancreatic cancer, and cytotoxicity in HeLa cells [37,107,108,109]. Moreover, a recent study reported the development of an MSN system co-loaded with DOX and β-elemene, a natural chemical compound, resulting in improved synergistic chemotherapy outcomes in esophageal cancer [110]. Accordingly, MSNs-DOX nanoconjugates exhibit increased bioavailability and therapeutic efficacy, paving the way for targeted cancer treatments and the development of personalized medicine [110]. The therapeutic index of cervical cancer treatment using MSNs has been further enhanced through the co-delivery of cisplatin and aluminum chloride phthalocyanine (AlClPc) within the same MSN platform [111]. Interestingly, multifunctional MSNs delivering paclitaxel together with tetrandrine in breast cancer cells also showed promising results, effectively overcoming tumor MDR [112]. In an effort to treat advanced or metastatic breast cancers, El-Shahawy et al. took advantage of the unique properties of MSNs and designed a theranostic nanoplatform comprised of magnetite nanoparticles with mesoporous silica, coated with chitosan. The aforementioned nanostructured system was further loaded with Abemaciclib, an inhibitor of cyclin-dependent kinases 4 and 6 [113]. Moreover, in a recent study, functionalized hollow MSNs were synthesized and utilized as nanocarriers for the antineoplastic agent fludarabine [114]. Specifically, fludarabine-loaded, alkylammonium-functionalized hollow MSNs significantly reduced MCF-7 cell viability in a pH-dependent manner, compared to the free drug [114]. Ultimately, in the context of breast cancer, the synergistic therapeutic effects of curcumin and silymarin incorporated in MSN have been outlined, while MSNs loaded with resveratrol have also been used for targeted cancer therapy, offering additional therapeutic options in this type of cancer [115,116]. 

Another promising approach for cancer treatment is photodynamic therapy (PDT) [97]. Silica-based nanoparticles have been widely explored as carriers in order to deliver the photosensitizers (PSs) directly to neoplastic cells and mitigate their limitations in clinical applications [117]. Depending on the type of PS used, various platforms have been developed, including porphyrin-loaded MSNs, phthalocyanine-loaded MSNs, chlorin e6-loaded MSNs, indocyanine green-loaded MSNs, and others [117]. Examples of such advances include the design of silica-PS conjugates for PDT against pancreatic cancer cells [118]; the development of 3D dendritic mesoporous silica nanoparticles for the delivery of novel hydrophobic photosensitizers [119]; and the engineering of dendritic mesoporous organosilica nanoparticles (MONs) capable of encapsulating indocyanine green (ICG) photosensitizer on macromolecular catalase (CAT) in order to overcome hypoxia of tumor cells and enhance PTD therapy [120]. In line with these strategies, Makhadmeh et al. encapsulated temoporfin (porphyrin derivative) in silica nanoparticles, enabling its controlled release and reducing off-target toxicity, thereby providing insights into efficient breast cancer therapy [121]. Intriguingly, silica nanoparticles have also been explored as potential platforms for dual chemo-photodynamic treatment, exhibiting promising results in human breast (MCF-7) and lung (A549) cancer lines [122]. 

Gene delivery has gained considerable attention in cancer therapy, as it enables the transfer of therapeutic genes into a cancer cell or tissue [123]. The incorporation of positively charged groups (amines, quaternary ammonium and guanidine groups) on the surface of MSNs favors the binding of negatively charged DNA or small interfering RNA (siRNA) molecules [124]. Leveraging these features, multiple studies have designed surface-functionalized MSNs for gene delivery in cancer [124]. For instance, MSN23 designed by Kim et al., is characterized by very large pores (>15 nm) and efficiently delivers siRNAs to the target and silences vascular endothelial growth factor (VEGF) in in vivo MDA-MB-231 xenografts [125]. Further research indicates that MSN platforms can simultaneously deliver DOX and Bcl-2 siRNAs, targeting Bcl-2 mRNAs, thereby triggering apoptosis and improving chemotherapy outcomes [126]. Tumor growth inhibition can also be achieved by delivering tumor suppressor genes, such as TP53, or siRNAs that silence oncogenes, suppressing cancer cell proliferation in lung and breast cancer [124]. Regarding breast cancer, human epidermal growth factor receptor 2 (HER2), an established oncogene responsible for this type of malignancy, has also been recognized as a therapeutic target. Thus, MSNs carrying siRNAs that knock down HER2 have been developed to enhance the existing therapeutic strategies against breast cancer [127]. In addition, P-glycoprotein (Pgp), which is overexpressed in drug-resistant breast cancer cells, drove Meng et al. to develop MSNs for the protected delivery of stably bound DOX and Pgp siRNA to the tumor site [128]. Given that oncogenic minibrain-related kinase (Mirk) and polo-like kinase 1 (PLK1) represent validated therapeutic targets, polylysine-functionalized large-pore mesoporous silica nanoparticles (MSNs) have been demonstrated to effectively deliver siRNAs directed against these kinases in osteosarcoma cells [129]. Ultimately, additional types of nucleic acids can be delivered through MSN complexes. In particular, tumor-suppressive miR-34a and anti-sense oligonucleotides targeting the oncogenic miR-21 can selectively be de-livered resulting in a decrease of neuroblastoma growth tumors and breast cancer cells, respectively [130,131]. 

In the past decade, immunotherapy, which is used to boost natural defenses to eliminate malignant cells, has emerged as a powerful strategy for treating numerous forms of cancer [132]. Notably, considering their unique properties, MSNs can significantly enhance cancer immunotherapy [133]. In brief, several research groups have combined MSN-based cancer vaccines with conventional chemotherapeutics such as DOX [134]. Furthermore, such platforms could be co-delivered with checkpoint inhibitors to block immune checkpoint proteins, the “brakes” of the immune system, thereby enabling more effective targeting and elimination of malignant cells [134]. Lastly, MSNs not only act as adjuvants, inducing both humoral and cell-mediated immune responses, but also serve as antigen carriers and immunomodulatory scaffolds [133].

### 3.9. Superiority of Alumina and Silica Substrates over Similar Types of Nanoparticles and Traditional Methods

Over the years, several different nanoplatforms have been constructed and studied regarding their potential applications in biosensing as well as drug delivery. Some well-known platforms are nanogels and lipidic nanocarriers. Polymeric nanocarriers offer great control over specific target detection and site-selective drug release [135]. Nanogels’ highly tunable properties offer substantial flexibility in terms of applications in theranostics [136]. They display key characteristics that allow for situational properties’ modification, such as size, charge, architecture and softness, based on the needs [137]. Lipid-based nanoplatforms, such as micelles, due to their hydrophobic properties and their ability to mimic organic structures, can carry a variety of payloads into cells. A great application of said carriers has been the mRNA vaccines, especially after COVID-19 [138]. However, their structural integrity is not very stable. These platforms possess incomparable attributes; however, alumina- and silica-based nanoplatforms offer improved characteristics with minimal trade-offs, mainly nano-bio unwanted immune reactions. Silica nanoplatforms offer high tunability of pore size and surface area, like nanogels, but with the advantage of more robust architectural chemistry, allowing for a greater variety of possible attachments and structures, offering them high versatility and enhanced uptake potential by the target cells/tissues [139,140] along with low toxicity. Alumina platforms also offer high versatility since they combine structural rigidity and a large modifiable surface. They can be constructed via the use of different techniques that result in different properties (pore size, architecture, etc.). Apart from their use in tissue repair, such as bone reformation, their cytotoxic properties against cancer cells and their low toxicity in normal tissues, along with their aforementioned characteristics, make alumina nanoplatforms ideal for pathology detection and cancer treatment.

Similarly, several techniques have been developed over the years to cover the needs of cancer diagnosis. Methods such as enzyme-linked immunosorbent assay (ELISA) and polymerase chain reaction (PCR) offer detection of cancer markers and genes in a variety of biological samples, have the benefit of detecting cancer-related material in low concentrations and are also easy to perform. Nevertheless, alumina- and silica-based nanoplatforms offer all the advantages of the aforementioned techniques while omitting several of their limitations. Characteristically, their porous structure makes them the perfect candidate for pathological marker detection, since they can encapsulate a variety of detection-mediating molecules. Furthermore, their characteristics allow them to detect markers with higher efficacy than already established, commercially available kits, down to the pico- and femto-per-ml scale and even detect nucleic acids without the need of copy amplification. Apart from greater detection range, these nanoplatforms offer extremely fast run-times from a few minutes down to a couple of seconds. Along with these perks that make them highly adaptable, alumina and silica platforms also offer immense structural robustness (some being stable for over 150 days) retaining their sensitivity, combined with low toxicity and potential use for in vivo imaging [49,141,142,143].

## 4. Understanding the Immune Interactions and Biodistribution of Alumina and Silica Substrates Toward Clinical Translation

**Figure 6:**
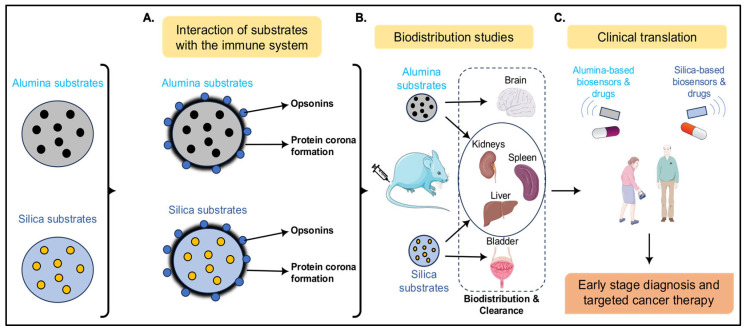
Immune interactions, biodistribution, and clinical translation of alumina- and silica-based nanoplatforms. Once alumina- and silica-based nanoplatforms enter the circulation, they interact with immune components through protein corona formation and opsonization (**A**), which govern their in vivo biodistribution and clearance, with primary accumulation and clearance involving the liver, spleen, kidneys, brain, and bladder (**B**). Ultimately, these processes critically influence the translational and clinical potential of cancer theranostic platforms (**C**). “Mouse and human icons were provided by Servier Medical Art (https://smart.servier.com/), licensed under CC BY 4.0 (https://creativecommons.org/licenses/by/4.0/)”.

Toward future clinical translation, it is crucial to understand the immunological interactions of alumina- and silica-based substrates. Once these nanoparticles enter the circulation, they rapidly engage with components of the immune system, and their biological fate depends on several factors such as protein corona formation, opsonization and subsequent macrophage uptake (Figure 6).

In this context, alumina nanoplatforms interact strongly with the immune guardians through protein adsorption and immune recognition. These particles rapidly ac-quire a protein-rich corona, a dynamic layer of proteins that adsorb around nanoparticles from biological fluids, and their surface functionalization influences the composition of the adsorbed proteins upon exposure to biological fluids [144]. Additionally, opsonization (the deposition of complement proteins on the surface of nanoparticles) of alumina-based platforms enhances their recognition and uptake by macrophages, thus modulating immune responses and amplifying their adjuvant effects [145]. In greater detail, this implies that immune activation can be achieved from the inorganic alumina lattice itself rather than from the therapeutic payload. Mechanistically, such alum-mediated adjuvanticity arises from particle internalization and the subsequent phagolysosomal destabilization and lysosomal rupture [102,146]. The latter leads to cathepsin release, and consequently to the activation of the NLRP3 inflammasome, leading to the caspase-1-dependent secretion of the pro-inflammatory cytokines IL-1β and IL-18, independently of the loaded cargo [147].

Similarly, multiple studies highlight the significant influence of protein corona formation in shaping the functionality of silica nanoparticles, thus modulating cellular interactions and immunological responses. Clemments et al., synthesized mesoporous silica particles with a range of diameters, showing that the composition of protein corona depends strongly on particle porosity and characteristics [147]. Additional studies in macrophages demonstrate that protein corona formation mitigates toxicity for all silica nanoparticles regardless of size [148]. More recently, an independent group reported that plasma protein adsorption onto MSNs regulates immune signaling pathways, highlighting the importance of corona formation and composition in regulating host and silica-based nanomaterials interactions [149]. Furthermore, protein corona formation is a crucial factor controlling the differential uptake of silica nanoparticles by human M1 macrophages and M2 polarized macrophages [150]. In parallel, silica substrates can also intrinsically activate immune signaling pathways. In particular, multiple studies that support that silica-induced cellular stress promotes the release of host-derived self-DNA, including mitochondrial DNA, into the cytoplasm, thereby activating the cGAS-STING pathway and downstream type I interferon signaling [151]. Additionally, silica-based nanomaterials have been reported to indirectly modulate toll-like receptor (TLR)-associated signaling. For example, a recent study in macro-phages pre-exposured to silica nanoparticles supports alterations in TLR4 localization, thereby influencing endolysosomal trafficking and NF-κB activation following cellular uptake [152].

The biodistribution of alumina and silica substrates is strongly influenced by the patient-specific nature of protein corona formation. To enable effective translation into clinical studies, it is therefore crucial to understand the dynamic evolution of the protein corona formation across different patient backgrounds. Key biological parameters, including biological sex, genetic ancestry, disease state, environment and biological age, significantly affect the pharmacokinetics and pharmacodynamics of these nanocarriers, thereby challenging the traditional “one-size-fits-all” strategy [153]. A commonly adopted strategy to mitigate immune clearance is the reduction of opsonization by making alumina and silica nanoparticles less visible to the immune system, thereby increasing their circulation half-life and potentially improving therapeutic efficacy [154]. In greater depth, methods targeting the attachment of polymer, protein, or bio-mimetic coatings have been developed and used as stealth agents on silica and alumina substrates. For example, polyethene glycol (PEG) is the most commonly used stealth agent, as it is water-soluble and enables extension of nanocarriers’ circulation half-life [155]. Beyond PEGylation, zwitterionic polymers (sulfobetaines, phosphorylcholine, and peptides) have also emerged as potential stealth agents, due to their similar behavior to PEG, enabling increased circulation in in vivo tumor-bearing nude mice [156]. However, in recent years, research has increasingly focused on biologically derived coatings, where the incorporation of hydroxyethyl starch (HES) onto the surface of alumina- and silica-based substrates can efficiently reduce their clearance [156]. Furthermore, an alternative strategy for the construction of stealth nanoparticles involves a priori design of protein coatings (dysopsonins, albumin and apolipoproteins) and bio-mimetic coronas (blood cell proteins and patient-derived proteins) to deliberately tune or exploit the protein corona for enhanced targeted delivery and sneak past the immune system’s defenses [157,158,159]. In contrast to the above stealth agents, several studies report that coating silica nanoparticles with poly(2-methyl-2-oxazoline) (PMOXA) does not prevent immune recognition, as it triggers C1q-mediated complement activation and C3 opsonization, leading to phagocytic clearance [160].

The immune-mediated interactions directly influence the biodistribution and clearance of alumina- and silica-based nanoplatforms, which are strongly size- and design-dependent (Figure 6) [11]. Alumina nanoparticles (AlNPs) exhibit efficient systemic absorption and predominantly accumulate in reticuloendothelial system (RES) organs, most notably the liver and spleen, with additional retention in the kidneys and, in certain models, the brain [161]. In vivo toxicological assessments of anodic alumina nanotubes further indicate a route-dependent biodistribution profile: while intravenous administration leads to accumulation in the liver and spleen due to RES sequestration, subcutaneous implantation facilitates local clearance through active immune cell infiltration [162]. Despite the growing body of data describing the short-term biodistribution of alumina-based nanoplatforms, systematic long-term, multi-month studies remain an ongoing challenge. Consequently, as the majority of available in vivo investigations are limited to short-term observations, the ultimate fate of alumina nanostructures beyond the therapeutic window remains insufficiently understood. Although persistent accumulation in RES has been reported, as discussed above, it remains unclear whether this reflects permanent sequestration or gradual, slow clearance.

In parallel, silica nanoparticles display biodistribution profiles that are strongly dependent on particle size and surface properties [163]. Following systemic administration, ultrasmall silica nanoparticles (<5–6 nm) are efficiently cleared via renal excretion in human imaging studies, whereas larger particles predominantly accumulate in RES organs, exhibiting prolonged hepatic and splenic retention [164]. Notably, the biodistribution and biodegradation of silica nanostructures vary with physicochemical properties, including particle geometry and shape, raising organ-specific clearance and safety considerations [163]. Regarding long-term biodistribution, in vivo studies in mice have reported up to 8 weeks persistence of silica nanoparticles in RES organs, raising concerns about their bioaccumulation over time [165]. Furthermore, the kinetics of in vivo degradation and clearance of silica nanomaterials are strongly influenced by design parameters. However, comparative long-term clearance profiles as a function of pore geometry are currently lacking. As noted above, existing literature predominantly interrogates short-term biodistribution for both alumina and silica substrates regarding geometry-dependent clearance and persistence. Collectively, these findings highlight the need for long-term biodistribution and clearance studies spanning several months, coupled with pharmacokinetic and histopathological analyses on alumina- and silica-based substrates.

Taken together, advancing alumina- and silica-based platforms toward clinical translation requires a thorough understanding of their safety, biodistribution and toxicological profiles, including ethical considerations related to long-term safety and patient risk (Figure 6). In the case of silica-based systems, limited but important progress has been made toward clinical studies. Interestingly, Philips et al. reported a first-in-human clinical trial employing renally excreted silica substrates radiolabeled with 124I for positron emission tomography (PET) imaging. This nanoplatform enables the detection of lesions, cancer staging and treatment options in patients with melanoma and brain cancer [166]. In addition, clinical studies have investigated gold shell-silica nanoparticles capable of generating heat for photothermal tumor ablation in head, neck and prostate cancers [167,168]. However, despite extensive preclinical data, the translation of silica-based nanoplatforms into further human clinical trials remains an open challenge due to various biological barriers. These include discrepancies between animal models and human physiology, which contributes to the existing gap between pre-clinical toxicological studies and patient safety outcomes [169]. Additional key biological hurdles that must be addressed for silica nanoparticles’ clinical translation include establishing safety following chronic exposure and comprehensive long-term toxicological profiles across different routes of administration [167]. Regarding alumina-based nanoparticles, despite the extensive and promising preclinical data, they have not yet advanced into clinical trials. Although alumina nanoparticles have shown antitumor efficacy in in vivo models, comprehensive pharmacokinetic and toxicity studies are lacking. Importantly, existing non-oncological in vivo studies provide valuable insights into their biodistribution and safety profile yet are insufficient to support clinical translation in cancer settings due to the bioaccumulation of inorganic materials and the potential immunogenicity and toxicity [170,171,172,173].

Beyond biological challenges, significant technological barriers should be addressed before the clinical translation of alumina- and silica-based platforms. Despite the availability of sophisticated fabrication strategies (EASA, ALD and hierarchical pore engineering), which enable the precise control of particle size, pore structure and functionality, the progression beyond laboratory-scale systems remains challenging. In particular, achieving reproducible large-scale fabrication for clinical grade manufacturing still needs optimization in order to maintain the same pore size, surface area and particle morphology [174,175]. Furthermore, multiple studies highlight that the production of defect-free nanoplatforms, with tightly controlled physicochemical characteristics, still remains an open challenge, limiting their progression beyond proof-of-concept studies toward regulatory approval and broader clinical trials [176,177].

## 5. Conclusions and Future Directions

Since the emergence of nanomedicine in the 1990s, a great expansion of research in the field of cancer treatment has been achieved. In this review article, the biomedical applications of alumina and silica substrates in cancer have been discussed. The key features of these materials, including mechanical stability, tunable porosity, and high biocompatibility, highlight their potential as highly promising candidates in cancer theranostics. In particular, both alumina and silica have been used in order to develop highly sensitive platforms for the detection of numerous cancer biomarkers, including CA15-3, CD3, SAA1, HER2, and EpCAM. NAA platforms, owing to their highly organised pore design and strong optical interference effects, often achieve extremely low detection limits, ranging from femtogram (10^−15^ g/mL) to attogram (10^−18^ g/mL) range [178], thus making early-stage and label-free biomarker identification possible. In parallel, mesoporous silica-based systems offer enhanced chemical flexibility and support a wider variety of signal amplification techniques, enabling the recognition of numerous cancer biomarkers with great sensitivity. Importantly, material selection for biosensing platforms should not be guided by sensitivity alone; translational context, target analyte characteristics, and the intended sensing modality must also be carefully considered.

Taking all the aforementioned points into consideration, it is evident that both alumina- and silica-based systems constitute very innovative and promising platforms in the biomedicine field of cancer theragnostic. As is presented in Table 2, all the geometrical characteristics of alumina- and silica-based systems are tunable (size, shape, length, and pores). The latter serves as a cornerstone for their increased sensitivity and specificity, both in targeted drug loading and release. Furthermore, these traits, in conjunction with their easy modification of functionalization of their surface, are the basis for the creation of very sensitive biosensors. In line with this notion, alumina and silica systems demonstrate increased stability and biocompatibility while their cytotoxicity is very low, both in vivo and in vitro. Of note, there are some features that differentiate between the two systems, such as drug-loading capacity. Particularly, silica-based systems can carry larger amounts of the drug than alumina, and they are characterized by enhanced tumor permeability. Regarding their respective limitations, each system appears to offset the shortcomings of the other. For example, silica-based systems are injected intravenously, which may influence their movement through the circulation. Moreover, their silanol groups promote hemolysis, both drawbacks that alumina-based systems fully overcome. Lastly, the enzyme-based biosensing systems for both of them present difficulties in enzyme encapsulation, which renders them very sensitive. All in all, both alumina- and silica-based systems exert multiple and unique advantages in comparison to traditional biomarkers and other nanoparticles, while their disadvantages are very limited and possible to overcome in the future.

As long as it concerns the restricted loading capacity of NAA, their pore properties can be conveniently tailored by changing the parameters of anodization. The latter have been exploited for creating NAAs for a plethora of applications in order to load large molecules. More specifically, a significant progress toward NAA fabrication with wider pore ordering was accomplished by Masuda and Fukuda, which changed this material growth to a substantial degree. Their simple electrochemical method uses a two-step anodization protocol in order to achieve the fabrication of highly ordered nanostructures that rely on the self-ordering phenomenon. All in all, the geometrical characteristics such as pore dimensions, size, shape, interpore distance, and length can be controlled by modifying anodization voltage, type, electrolyte concentration, and temperature [76,179].

Apart from the enormous potential for next-generation bioanalysis, multifunctional alumina- and silica-based nanostructures have been developed, addressing challenges of conventional cancer treatments, including drug toxicity and resistance, as well as tumor heterogeneity. For instance, the incorporation of chemotherapeutics that are used in everyday clinical practice (e.g., doxorubicin), combined with other compounds, has shown improved bioavailability and therapeutic efficacy. Furthermore, a plethora of recent advances in nanoporous anodic alumina refer to the creation of sensing platforms based on the interaction of oligonucleotides and antibodies with bioelements that can help in disease detection and monitoring in current oncology. More specifically, nucleic acids can bind to distinct elements such as small organic molecules, proteins, or cells with increased selectivity, specificity, and affinity, even higher than those of antibodies [17]. The fabrication of biosensors for cancerous mutation detection has gained increased interest. DNA sensors have been manufactured with NAA oligonucleotide modified substrates using several detection techniques: voltammetry, impedimetric sensing, RIfS, fluorescence and LSPR. The aforementioned traits prove the crucial role of alumina-based platforms in the nucleic-acid-based therapeutics that dominate current oncology pipelines. Another promising application of alumina and silica substrates is associated with the detection and elimination of senescent cells, which are increasingly recognized as contributors to cancer progression and therapy resistance [180,181,182]. In this context, the combinatorial administration of chemotherapeutics and senolytics using these platforms may prevent tumor relapses, expanding patients’ health span [183].

Cancer treatment requires the integration of multiple therapeutic modalities to effectively address tumor complexity and overcome resistant mechanisms. Therefore, the combination of alumina- and silica-based nanoplatforms with other types of treatments such as radiotherapy [184], hyperthermia [185], pulsed electromagnetic field stimulation (PEMFs) [186] and targeted molecular therapies [187] could be proven to be highly advantageous. Based on their unique properties, both alumina and silica materials have also shown encouraging outcomes in infectious diseases, Alzheimer’s disease and various metabolic pathologies (e.g., diabetes, osteoporosis, etc.) [188]. To conclude, these materials are poised to contribute substantially to the cure of cancer and other complex diseases, ultimately supporting the development of more effective therapies and improving patient survival and quality of life.

Despite their potential in controlled drug delivery and nanomedicine, a significant translation gap exists between lab-scale fabrication techniques and industrial-scale manufacturing. Maintaining precise batch-to-batch repeatability of nanopore dimensions, pore ordering, and surface chemistry is challenging, but extremely necessary as every technical detail can have an immense impact on drug loading efficiency, release kinetics, and biological interactions. Furthermore, when compared to clinically approved and industrially used platforms such as liposomal formulations, alumina and silica-based systems frequently face economic and practical constraints, such as high production costs, more complex quality control requirements, and, most importantly, less established regulatory pathways [189,190]. These factors, taken together, restrict large-scale industrial manufacturing, emphasizing the importance of scalable fabrication processes and rigorous techno-economic evaluations to support clinical translation.

Future research on alumina- and silica-based platforms should increasingly focus on bridging the gap between material design and specific oncological applications, moving beyond descriptive demonstrations toward rational, function-oriented engineering. In line with the synthesis and functionalization strategies discussed, key research hotspots are expected to involve the development of stimuli-responsive and multifunctional theranostic systems, in which pore architecture, surface chemistry, and material composition are deliberately optimized to match the requirements of biosensing, drug delivery, or combined applications [191]. Specifically, the development of highly ordered optical and electrochemical biosensors, implantable diagnostic devices, and mechanically defined substrates for studying tumor cells behavior and microenvironmental interactions can be significantly supported by nanoporous anodic alumina platforms. At the same time, silica-based systems, particularly mesoporous and dendritic architectures, have the potential to accelerate future advancements by designing large-pore, surface-functionalized carriers that can successfully load and release chemotherapeutics, photosensitizers, and nucleic acid-based therapeutics, thus enabling increasingly complex combinatorial therapies [192]. However, despite the aforementioned advantages, the presence of some limitations, presented in Table 2, is still under investigation for the improvement of alumina- and silica-based nanoplatforms translated into clinically useful tools for cancer theranostics. Long-term research on biocompatibility, biodegradation, clearance, and immunological interactions is also ongoing, especially for hybrid and multifunctional platforms.

The current oncology theranostic landscape is characterized by multi-marker approaches for cancer detection and treatment. Liquid biopsies serve as a cornerstone in multi-marker cancer diagnostics since they offer a sensitive, specific, cost-effective and non-invasive alternative to the conventional surgical tumor biopsy. To this day, the majority of alumina- and silica-based biosensors, utilized for liquid sample analysis, are designed for single-analyte detection, such as well-established tumor biomarkers commonly used in cancer diagnostics (e.g. CA15-3, SAA1, EpCAM, HER2) [49]. However, there is growing recognition that multiomic biosensing approaches capable of simultaneously interrogating DNA, RNA, and protein biomarkers are essential to better capture tumor heterogeneity from liquid biopsies. Tumor evolution is characterized by constant genetic, transcriptomic, proteomic and metabolomic alterations that can be identified through multiomic analysis, leading to personalized early diagnosis and effective treatment. Due to their tunability in size, capacity and number of pores, alumina and silica substrates constitute ideal scaffolds for the immobilization and spatial organization of multiple biosensing systems. Additionally, as it has already been highlighted, alumina and silica nanomaterials exhibit high biocompatibility and low toxicity, thereby offering a further critical advantage for their integration into biosensing platforms intended for biological fluids and clinical diagnostic applications. Although multiomic biosensors conjugated on alumina or silica substrates remain at an early stage of development, recent advances in microfluidics, surface patterning, and signal multiplexing suggest that such platforms are technically feasible and represent a compelling future direction for next-generation cancer diagnostics.

Nanoporous anodic alumina has mostly been studied in the field of materials science and biosensing [193]. Although these studies offer valuable insights, they typically examine silica and alumina substrates independently, while they solely focus on their therapeutic and diagnostic applications [194]. In contrast, this review integrates silica- and alumina-based systems within a unified theranostic framework, providing a comprehensive overview alongside a critical analysis of synthesis strategies, surface functionalization methods, advantages and limitations, and their corresponding biological applications on biosensing and drug delivery.

## Figures and Tables

**Table 1:** Different functionalization methods of alumina and silica substrates.

Functionalization Method	Materials Used	Technique/Process	Main Effect	Application in Cancer Theranostics/Biomedicine	Substrate
**Metal Deposition**	Gold (Au), silver (Ag), Platinum (Pt), Palladium (Pd), Titanium (Ti), Nickel (Ni), Cobalt (Co)	Sputtering, Evaporation, Chemical Vapor Deposition (CVD)	Improves conductivity, catalytic activity, antimicrobial properties	Drug delivery platforms, biosensors, therapeutic coatings	Alumina
**Silver Modification**	Silver (Ag)	Metal coating	Rejuvenates fibroblast cells	Tissue regeneration, cancer-related implant research	Alumina
**Polymer Electropolymerization**	Polypyrrole (PP)	Electropolymerization inside pores → nanowire formation	Alters surface topography, enhances cell adhesion	Cell–material interaction control, cancer scaffolds	Alumina
**Polymer Coating**	Polymethylmethacrylate (PMMA)	Electrophoretic Deposition (EPD)	Increases ALP enzymatic activity and osteoconductivity	Bone cancer research, implant-related theranostics	Alumina
**Chemical Surface Functionalization**	APTES, Phosphonic acids	Silanization, Phosphonic acid grafting	Enables selective molecular binding	Drug loading, biosensing, microfluidics	Alumina
**Thin Film Coating**	Various metal and oxide thin films	Atomic Layer Deposition (ALD), Sol–gel modification	Precise nanoscale surface control	Drug reservoirs, antibody platforms, biosensors	Alumina
**Co-condensation**	Organic and inorganic groups, antimicrobial compounds, small biomolecules, or ions	Functionalizing group is added to the sol–gel solution	Homogeneous distribution of functionalizing	For chromatographic applications and drug delivery	Silica
**Post-grafting**	Organic and inorganic groups, antimicrobial compounds, small biomolecules, or ions	Functionalizing groups are attached to the mesoporous surface after the film consolidation	Alteration of surface properties	Ιmaging and drug delivery	Silica

**Table 2:** Unique properties of alumina and silica substrates for cancer theranostics.

	Properties	Alumina	Silica
**Advantages**	Tunable Geometrical Characteristics (size, shape, length)	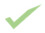	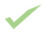
	Tunable Pore Size	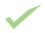	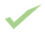
	High Stability	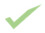	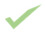
	Increased Biocompatibility	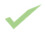	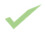
	High Drug Loading Capacity	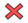	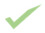
	Modification in Surface Functionalization	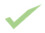	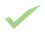
	Enhanced Tumor Permeability	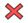	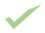
	Low in vitro and in vivo toxicity	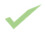	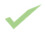
	Cost effective and large-scale production	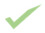	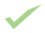
**Disadvantages**	Preferable Medication Route	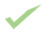	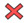
	Hemolysis	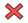	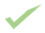
	Immune recognition and possible clearance	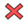	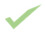
	Difficult enzyme encapsulation	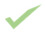	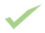
	Restricted Movement through the circulation	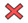	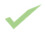

## Data Availability

No new data were created or analyzed in this study. Data sharing is not applicable to this article.

## Abbreviations

The following abbreviations are used in this manuscript:

8-oxo-dG	8-oxo-7,8-dihydro-2′-deoxyguanosine
ABTS	2,2′-azino-bis(3-ethylbenzothiazoline-6-sulfonic acid)
ACP	acid phosphatase
Al_2_O_3_	aluminum oxide
Al_2_O_3_NPs	alumina nanoparticles
AlClPc	aluminum chloride phthalocyanine
ALD	atomic layer deposition
ALP	alkaline phosphatase
anti-EpCAM	epithelial cell adhesion molecule antibodies
APTES	(3-Aminopropyl)triethoxysilane
AuNPs	gold nanoparticles
BDMSNs	biphenyl-decorated mesoporous silica nanoparticles
CA15-3	cancer antigen 15-3
CA 19-9	cancer antigen 19-9
CAT	catalase
CEA	carcinoembryonic antigen
CpG	5′-C-phosphate-G-3′
CS	chitosan
CTCs	circulating tumor cells
CVD	chemical vapor deposition
CYFRA 21-1	cytokeratin fragment 19
DMSNs	dendritic mesoporous silica nanoparticles
DOX	doxorubicin
DTT	dithiothreitol
EASA	electro-assisted self-assembly
ECL	electrochemiluminescence
EISA	evaporation-induced self-assembly
ELISA	enzyme-linked immunosorbent assay
EPD	electrophoretic deposition
GPC1	glypican-1
GSH	glutathione
H_2_O_2_	hydrogen peroxide
HER2	human epidermal growth factor receptor 2
HMHAs	hyaluronic acid-coated mesoporous hollow alumina nanoparticles
ICG	indocyanine green
ITO	indium tin oxide
LOD	limit of detection
LRSP	localize surface plasmon resonance
MB	methylene blue
MDR	multidrug resistance
MIP	molecularly imprinted polymer
Mirk	minibrain-related kinase
MLFIA	multiplex lateral flow immunoassay
MONs	mesoporous organosilica nanoparticles
MSiO_2_	mesoporous silica particles
MSNs	mesoporous silica nanoparticles
MSF	mesoporous silica films
MTT	(3-(4,5-Dimethylthiazol-2-yl)-2,5-Diphenyltetrazolium Bromide)
NAA	nanoporous anodic alumina
PCR	polymerase chain reaction
PDT	photodynamic therapy
PEC	photoelectrochemical
Pgp	P-glycoprotein
PLK1	polo-like kinase 1
PMMA	polymethylmethacrylate
PPy	polypyrrole
PQQ	pyrroloquinoline quinone
PSA	prostate specific antigen
PSs	photosensitizers
PtNPs	platinum nanoparticles
PTT	photothermal therapy
PVP	polyvinylpyrrolidone
QC	quercetin
ROS	reactive oxygen species
SAA1	serum amyloid A1
SBMM	silica-based mesoporous material
SDAs	structure-directing agents
Si–O–Si	siloxane
Si-OH	silanol groups
SiO_2_-PEI	polyethylenimine-modified silica
siRNA	small interfering RNA
VEGF	vascular endothelial growth factor
VMSFs	vertically ordered mesoporous silica films
